# 5-Bromo-3-(4-fluoro­phenyl­sulfin­yl)-2,4,6-trimethyl-1-benzofuran

**DOI:** 10.1107/S1600536811002303

**Published:** 2011-01-22

**Authors:** Hong Dae Choi, Pil Ja Seo, Byeng Wha Son, Uk Lee

**Affiliations:** a Department of Chemistry, Dongeui University, San 24 Kaya-dong Busanjin-gu, Busan 614-714, Republic of Korea; b Department of Chemistry, Pukyong National University, 599-1 Daeyeon 3-dong, Nam-gu, Busan 608-737, Republic of Korea

## Abstract

In the title mol­ecule, C_17_H_14_BrFO_2_S, the 4-fluoro­phenyl ring makes a dihedral angle of 75.92 (5)° with the mean plane of the benzofuran fragment. In the crystal, mol­ecules are linked by weak inter­molecular C—H⋯O hydrogen bonds and aromatic π–π inter­actions between the benzene and the furan rings of neighbouring mol­ecules [centroid–centroid distance = 3.556 (1) Å].

## Related literature

For the pharmacological activity of benzofuran compounds, see: Aslam *et al.* (2006 ▶); Galal *et al.* (2009 ▶); Khan *et al.* (2005 ▶). For natural products with benzofuran rings, see: Akgul & Anil (2003 ▶); Soekamto *et al.* (2003 ▶). For our previous structural studies of related 5-bromo-3-(4-fluoro­phenyl­sulfin­yl)-2-methyl-1-benzofuran derivatives, see: Choi *et al.* (2010 ▶, 2011 ▶).
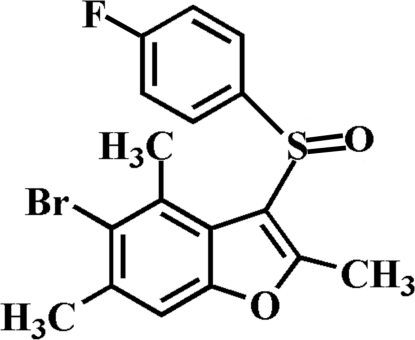


## Experimental

### 

#### Crystal data

C_17_H_14_BrFO_2_S
*M* *_r_* = 381.25
Triclinic, 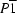

*a* = 7.6121 (3) Å
*b* = 8.3551 (3) Å
*c* = 13.0681 (4) Å
α = 71.551 (2)°
β = 84.206 (2)°
γ = 74.850 (2)°
*V* = 760.86 (5) Å^3^
*Z* = 2
Mo *K*α radiation
μ = 2.85 mm^−1^
*T* = 173 K
0.29 × 0.28 × 0.18 mm

#### Data collection

Bruker SMART APEXII CCD diffractometer
Absorption correction: multi-scan (*SADABS*; Bruker, 2009 ▶) *T*_min_ = 0.621, *T*_max_ = 0.746
13138 measured reflections
3491 independent reflections
3041 reflections with *I* > 2σ(*I*)
*R*_int_ = 0.020

#### Refinement

*R*[*F* ^2^ > 2σ(*F* ^2^)] = 0.026
*wR*(*F* ^2^) = 0.070
*S* = 1.03
3491 reflections
202 parameters
H-atom parameters constrained
Δρ_max_ = 0.37 e Å^−3^
Δρ_min_ = −0.36 e Å^−3^

### 

Data collection: *APEX2* (Bruker, 2009 ▶); cell refinement: *SAINT* (Bruker, 2009 ▶); data reduction: *SAINT*; program(s) used to solve structure: *SHELXS97* (Sheldrick, 2008 ▶); program(s) used to refine structure: *SHELXL97* (Sheldrick, 2008 ▶); molecular graphics: *ORTEP-3* (Farrugia, 1997 ▶) and *DIAMOND* (Brandenburg, 1998 ▶); software used to prepare material for publication: *SHELXL97*.

## Supplementary Material

Crystal structure: contains datablocks global, I. DOI: 10.1107/S1600536811002303/su2248sup1.cif

Structure factors: contains datablocks I. DOI: 10.1107/S1600536811002303/su2248Isup2.hkl

Additional supplementary materials:  crystallographic information; 3D view; checkCIF report

## Figures and Tables

**Table 1:** Hydrogen-bond geometry (Å, °)

*D*—H⋯*A*	*D*—H	H⋯*A*	*D*⋯*A*	*D*—H⋯*A*
C14—H14⋯O2^i^	0.95	2.35	3.232 (2)	154

Symmetry code: (i) 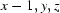.

**Fig. 1.**
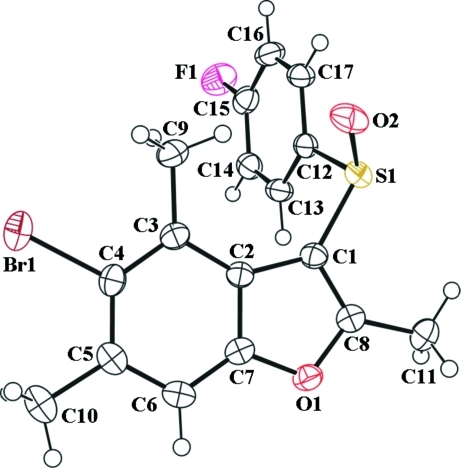
The molecular structure of the title compound with the atom numbering scheme. Displacement ellipsoids are drawn at the 50% probability level. H atoms are presented as a small spheres of arbitrary radius.

**Fig. 2.**
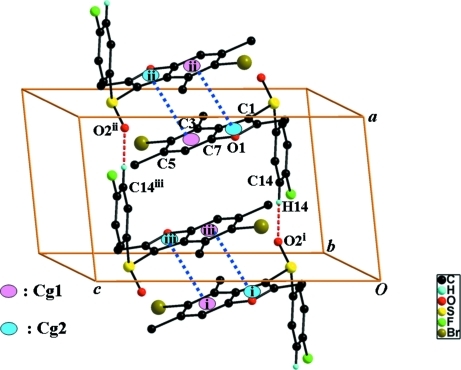
A view of the C—H···O and π–π interactions (dotted lines) in the crystal structure of the title compound [Cg1 and Cg2 are the centroids of the C2-C7 benzene ring and the C1/C2/C7/O1/C8 furan ring, respectively; Symmetry codes: (i) x - 1, y, z; (ii) - x + 2, - y + 1, - z + 1; (iii) - x + 1, - y + 1, - z + 1].

## supplementary crystallographic information

### Comment

Many compounds containing a benzofuran ring show interesting potent pharmacological properties, such as antifungal, antitumor and antiviral, and antimicrobial activities (Aslam *et al.*, 2006; Galal *et al.*, 2009; Khan *et al.*, 2005). These compounds occur in a wide range of natural products (Akgul & Anil, 2003; Soekamto *et al.*, 2003). As a part of our ongoing study of the substituent effect on the solid state structures of 5-bromo-3-(4-fluorophenylsulfinyl)-2-methyl-1-benzofuran analogues (Choi *et al.*, 2010; 2011), we report herein on the crystal structure of the title compound.

In the title molecule (Fig. 1), the benzofuran unit is essentially planar, with a mean deviation of 0.004 (1)Å from the least-squares plane defined by the nine constituent atoms. The dihedral angle formed by the mean plane of the benzofuran fragment and the 4-fluorophenyl ring is 75.92 (5)°.

In the crystal the molecular packing (Fig. 2) is stabilized by weak intermolecular C—H···O hydrogen bonds between the 4-fluorophenyl H-atom and the oxygen of the S═O unit (Table 1; C14–H14···O2^i^). The crystal packing (Fig. 2) is further stabilized by aromatic π–π interactions between the benzene and furan rings of the adjacent molecules. The Cg1···Cg2^ii^ distance of 3.556 (1)Å (Cg1 and Cg2 are the centroids of the C2-C7 benzene ring and the C1/C2/C7/O1/C8 furan ring, respectively; symmetry code: (ii) = - *x* + 2, - *y* + 1, - *z* + 1).

### Experimental

77% 3-chloroperoxybenzoic acid (224 mg, 1.0 mmol) was added in small portions to a stirred solution of 5-bromo-3-(4-fluorophenylsulfanyl)-2,4,6-trimethyl-1-benzofuran (329 mg, 0.9 mmol) in dichloromethane (30 mL) at 273 K. After being stirred at room temperature for 4h, the mixture was washed with saturated sodium bicarbonate solution and the organic layer was separated, dried over magnesium sulfate, filtered and concentrated at reduced pressure. The residue was purified by column chromatography (hexane–ethyl acetate, 4:1 v/v) to afford the title compound as a colorless solid [yield 73%, m.p. 424–425 K; *R*_f_ = 0.54 (hexane–ethyl acetate, 4:1 v/v)]. Single crystals, suitable for X-ray diffraction, were prepared by slow evaporation of a solution of the title compound in acetone at room temperature.

### Refinement

All the H-atoms were positioned geometrically and refined using a riding model: C–H = 0.95 Å for aryl and 0.98 Å for methyl H-atoms, with *U*_iso_(H) =1.2*U*_eq_(C) for aryl and 1.5*U*_eq_(C) for methyl H-atoms.

### Crystal data

**Table:**

C_17_H_14_BrFO_2_S	*Z* = 2
*M**_r_* = 381.25	*F*(000) = 384
Triclinic, *P*1	*D*_x_ = 1.664 Mg m^−^^3^
Hall symbol: -P 1	Mo *K*α radiation, λ = 0.71073 Å
*a* = 7.6121 (3) Å	Cell parameters from 6469 reflections
*b* = 8.3551 (3) Å	θ = 2.7–27.4°
*c* = 13.0681 (4) Å	µ = 2.85 mm^−^^1^
α = 71.551 (2)°	*T* = 173 K
β = 84.206 (2)°	Block, colourless
γ = 74.850 (2)°	0.29 × 0.28 × 0.18 mm
*V* = 760.86 (5) Å^3^	

### Data collection

**Table:**

Bruker SMART APEXII CCD diffractometer	3491 independent reflections
Radiation source: rotating anode	3041 reflections with *I* > 2σ(*I*)
graphite multilayer	*R*_int_ = 0.020
Detector resolution: 10.0 pixels mm^-1^	θ_max_ = 27.6°, θ_min_ = 1.6°
φ and ω scans	*h* = −9→9
Absorption correction: multi-scan (*SADABS*; Bruker, 2009)	*k* = −10→10
*T*_min_ = 0.621, *T*_max_ = 0.746	*l* = −16→16
13138 measured reflections	

### Refinement

**Table:**

Refinement on *F*^2^	Primary atom site location: structure-invariant direct methods
Least-squares matrix: full	Secondary atom site location: difference Fourier map
*R*[*F*^2^ > 2σ(*F*^2^)] = 0.026	Hydrogen site location: difference Fourier map
*wR*(*F*^2^) = 0.070	H-atom parameters constrained
*S* = 1.03	*w* = 1/[σ^2^(*F*_o_^2^) + (0.0383*P*)^2^ + 0.249*P*] where *P* = (*F*_o_^2^ + 2*F*_c_^2^)/3
3491 reflections	(Δ/σ)_max_ = 0.001
202 parameters	Δρ_max_ = 0.37 e Å^−^^3^
0 restraints	Δρ_min_ = −0.36 e Å^−^^3^

### Special details

**Table:**

Geometry. All esds (except the esd in the dihedral angle between two l.s. planes) are estimated using the full covariance matrix. The cell esds are taken into account individually in the estimation of esds in distances, angles and torsion angles; correlations between esds in cell parameters are only used when they are defined by crystal symmetry. An approximate (isotropic) treatment of cell esds is used for estimating esds involving l.s. planes.
Refinement. Refinement of F^2^ against ALL reflections. The weighted R-factor wR and goodness of fit S are based on F^2^, conventional R-factors R are based on F, with F set to zero for negative F^2^. The threshold expression of F^2^ > 2sigma(F^2^) is used only for calculating R-factors(gt) etc. and is not relevant to the choice of reflections for refinement. R-factors based on F^2^ are statistically about twice as large as those based on F, and R- factors based on ALL data will be even larger.

### Fractional atomic coordinates and isotropic or equivalent isotropic displacement parameters (Å^2^)

**Table:**

	*x*	*y*	*z*	*U*_iso_*/*U*_eq_	
Br1	0.68278 (3)	0.91822 (3)	0.615760 (17)	0.04054 (8)	
S1	0.95005 (6)	0.77098 (6)	0.16447 (4)	0.02943 (11)	
F1	0.29820 (17)	1.33971 (15)	0.03005 (12)	0.0499 (3)	
O1	0.81454 (17)	0.38324 (15)	0.38631 (10)	0.0286 (3)	
O2	1.09273 (18)	0.84985 (19)	0.18473 (12)	0.0412 (3)	
C1	0.8798 (2)	0.6334 (2)	0.28464 (14)	0.0245 (3)	
C2	0.8185 (2)	0.6538 (2)	0.38953 (14)	0.0231 (3)	
C3	0.7943 (2)	0.7847 (2)	0.43920 (15)	0.0255 (4)	
C4	0.7266 (2)	0.7438 (2)	0.54461 (15)	0.0273 (4)	
C5	0.6871 (2)	0.5839 (2)	0.60321 (15)	0.0290 (4)	
C6	0.7165 (2)	0.4558 (2)	0.55291 (16)	0.0299 (4)	
H6	0.6936	0.3450	0.5895	0.036*	
C7	0.7801 (2)	0.4951 (2)	0.44808 (15)	0.0252 (4)	
C8	0.8752 (2)	0.4705 (2)	0.28794 (15)	0.0267 (4)	
C9	0.8414 (3)	0.9549 (2)	0.38215 (17)	0.0351 (4)	
H9A	0.7298	1.0449	0.3573	0.053*	
H9B	0.9218	0.9435	0.3200	0.053*	
H9C	0.9035	0.9875	0.4318	0.053*	
C10	0.6144 (3)	0.5493 (3)	0.71775 (17)	0.0402 (5)	
H10A	0.6073	0.4276	0.7465	0.060*	
H10B	0.4927	0.6256	0.7191	0.060*	
H10C	0.6959	0.5720	0.7621	0.060*	
C11	0.9167 (3)	0.3742 (3)	0.20722 (17)	0.0348 (4)	
H11A	0.9711	0.4421	0.1423	0.052*	
H11B	0.8041	0.3550	0.1884	0.052*	
H11C	1.0024	0.2618	0.2376	0.052*	
C12	0.7454 (2)	0.9425 (2)	0.13416 (14)	0.0257 (4)	
C13	0.5734 (2)	0.9108 (2)	0.15511 (15)	0.0289 (4)	
H13	0.5602	0.7970	0.1932	0.035*	
C14	0.4209 (3)	1.0452 (2)	0.12041 (15)	0.0309 (4)	
H14	0.3019	1.0261	0.1343	0.037*	
C15	0.4475 (3)	1.2070 (2)	0.06525 (16)	0.0334 (4)	
C16	0.6159 (3)	1.2419 (2)	0.04298 (16)	0.0352 (4)	
H16	0.6282	1.3559	0.0044	0.042*	
C17	0.7673 (3)	1.1072 (2)	0.07804 (15)	0.0304 (4)	
H17	0.8858	1.1275	0.0637	0.036*	

### Atomic displacement parameters (Å^2^)

**Table:**

	*U*^11^	*U*^22^	*U*^33^	*U*^12^	*U*^13^	*U*^23^
Br1	0.04469 (13)	0.04331 (13)	0.03878 (13)	−0.00562 (9)	−0.00095 (9)	−0.02358 (10)
S1	0.0305 (2)	0.0312 (2)	0.0273 (2)	−0.01179 (18)	0.00611 (18)	−0.00835 (18)
F1	0.0479 (7)	0.0343 (6)	0.0595 (8)	−0.0004 (5)	−0.0100 (6)	−0.0080 (6)
O1	0.0337 (7)	0.0233 (6)	0.0299 (7)	−0.0091 (5)	−0.0026 (5)	−0.0073 (5)
O2	0.0314 (7)	0.0458 (8)	0.0478 (9)	−0.0209 (6)	0.0053 (6)	−0.0088 (7)
C1	0.0236 (8)	0.0255 (8)	0.0251 (9)	−0.0081 (6)	−0.0015 (7)	−0.0065 (7)
C2	0.0202 (8)	0.0248 (8)	0.0249 (9)	−0.0072 (6)	−0.0015 (7)	−0.0068 (7)
C3	0.0239 (8)	0.0250 (8)	0.0288 (9)	−0.0073 (7)	−0.0038 (7)	−0.0076 (7)
C4	0.0247 (8)	0.0307 (9)	0.0285 (9)	−0.0038 (7)	−0.0036 (7)	−0.0132 (8)
C5	0.0228 (8)	0.0370 (9)	0.0251 (9)	−0.0063 (7)	−0.0024 (7)	−0.0065 (8)
C6	0.0293 (9)	0.0281 (9)	0.0293 (10)	−0.0095 (7)	−0.0033 (8)	−0.0019 (7)
C7	0.0235 (8)	0.0235 (8)	0.0285 (9)	−0.0052 (6)	−0.0054 (7)	−0.0067 (7)
C8	0.0254 (8)	0.0273 (8)	0.0281 (9)	−0.0064 (7)	−0.0037 (7)	−0.0083 (7)
C9	0.0463 (11)	0.0296 (9)	0.0351 (11)	−0.0175 (8)	0.0016 (9)	−0.0115 (8)
C10	0.0366 (10)	0.0530 (12)	0.0297 (10)	−0.0125 (9)	0.0059 (9)	−0.0113 (9)
C11	0.0390 (10)	0.0322 (9)	0.0369 (11)	−0.0072 (8)	−0.0012 (9)	−0.0168 (9)
C12	0.0334 (9)	0.0271 (8)	0.0203 (8)	−0.0123 (7)	0.0025 (7)	−0.0092 (7)
C13	0.0365 (10)	0.0282 (9)	0.0242 (9)	−0.0147 (7)	0.0005 (8)	−0.0057 (7)
C14	0.0337 (9)	0.0361 (10)	0.0272 (9)	−0.0138 (8)	0.0008 (8)	−0.0118 (8)
C15	0.0414 (10)	0.0300 (9)	0.0292 (10)	−0.0052 (8)	−0.0047 (8)	−0.0108 (8)
C16	0.0509 (12)	0.0249 (9)	0.0321 (10)	−0.0155 (8)	−0.0015 (9)	−0.0065 (8)
C17	0.0394 (10)	0.0314 (9)	0.0261 (9)	−0.0189 (8)	0.0031 (8)	−0.0095 (8)

### Geometric parameters (Å, °)

**Table:**

Br1—C4	1.9077 (18)	C9—H9A	0.9800
S1—O2	1.4922 (14)	C9—H9B	0.9800
S1—C1	1.7559 (18)	C9—H9C	0.9800
S1—C12	1.7990 (18)	C10—H10A	0.9800
F1—C15	1.363 (2)	C10—H10B	0.9800
O1—C8	1.364 (2)	C10—H10C	0.9800
O1—C7	1.379 (2)	C11—H11A	0.9800
C1—C8	1.357 (2)	C11—H11B	0.9800
C1—C2	1.450 (2)	C11—H11C	0.9800
C2—C7	1.395 (2)	C12—C17	1.384 (2)
C2—C3	1.404 (2)	C12—C13	1.387 (3)
C3—C4	1.390 (3)	C13—C14	1.384 (3)
C3—C9	1.499 (2)	C13—H13	0.9500
C4—C5	1.406 (3)	C14—C15	1.373 (3)
C5—C6	1.386 (3)	C14—H14	0.9500
C5—C10	1.508 (3)	C15—C16	1.371 (3)
C6—C7	1.372 (3)	C16—C17	1.381 (3)
C6—H6	0.9500	C16—H16	0.9500
C8—C11	1.482 (3)	C17—H17	0.9500
			
O2—S1—C1	111.31 (9)	H9A—C9—H9C	109.5
O2—S1—C12	106.48 (8)	H9B—C9—H9C	109.5
C1—S1—C12	99.80 (8)	C5—C10—H10A	109.5
C8—O1—C7	106.59 (13)	C5—C10—H10B	109.5
C8—C1—C2	107.38 (15)	H10A—C10—H10B	109.5
C8—C1—S1	118.89 (14)	C5—C10—H10C	109.5
C2—C1—S1	133.73 (13)	H10A—C10—H10C	109.5
C7—C2—C3	119.38 (16)	H10B—C10—H10C	109.5
C7—C2—C1	104.21 (15)	C8—C11—H11A	109.5
C3—C2—C1	136.40 (16)	C8—C11—H11B	109.5
C4—C3—C2	115.25 (15)	H11A—C11—H11B	109.5
C4—C3—C9	122.91 (16)	C8—C11—H11C	109.5
C2—C3—C9	121.84 (16)	H11A—C11—H11C	109.5
C3—C4—C5	125.19 (17)	H11B—C11—H11C	109.5
C3—C4—Br1	117.61 (13)	C17—C12—C13	120.84 (17)
C5—C4—Br1	117.20 (14)	C17—C12—S1	116.44 (14)
C6—C5—C4	118.23 (17)	C13—C12—S1	122.38 (13)
C6—C5—C10	119.74 (18)	C14—C13—C12	119.93 (16)
C4—C5—C10	122.03 (18)	C14—C13—H13	120.0
C7—C6—C5	117.35 (17)	C12—C13—H13	120.0
C7—C6—H6	121.3	C15—C14—C13	117.72 (17)
C5—C6—H6	121.3	C15—C14—H14	121.1
C6—C7—O1	124.64 (16)	C13—C14—H14	121.1
C6—C7—C2	124.57 (17)	F1—C15—C16	118.27 (17)
O1—C7—C2	110.78 (15)	F1—C15—C14	118.11 (17)
C1—C8—O1	111.03 (16)	C16—C15—C14	123.61 (18)
C1—C8—C11	133.38 (18)	C15—C16—C17	118.30 (17)
O1—C8—C11	115.56 (15)	C15—C16—H16	120.9
C3—C9—H9A	109.5	C17—C16—H16	120.9
C3—C9—H9B	109.5	C16—C17—C12	119.60 (17)
H9A—C9—H9B	109.5	C16—C17—H17	120.2
C3—C9—H9C	109.5	C12—C17—H17	120.2
			
O2—S1—C1—C8	−129.96 (14)	C8—O1—C7—C2	0.00 (18)
C12—S1—C1—C8	117.93 (14)	C3—C2—C7—C6	0.9 (3)
O2—S1—C1—C2	50.88 (19)	C1—C2—C7—C6	−179.68 (17)
C12—S1—C1—C2	−61.23 (18)	C3—C2—C7—O1	−179.17 (14)
C8—C1—C2—C7	−0.43 (18)	C1—C2—C7—O1	0.26 (18)
S1—C1—C2—C7	178.79 (15)	C2—C1—C8—O1	0.47 (19)
C8—C1—C2—C3	178.85 (19)	S1—C1—C8—O1	−178.89 (11)
S1—C1—C2—C3	−1.9 (3)	C2—C1—C8—C11	178.42 (19)
C7—C2—C3—C4	−2.0 (2)	S1—C1—C8—C11	−0.9 (3)
C1—C2—C3—C4	178.83 (18)	C7—O1—C8—C1	−0.30 (19)
C7—C2—C3—C9	177.13 (16)	C7—O1—C8—C11	−178.65 (15)
C1—C2—C3—C9	−2.1 (3)	O2—S1—C12—C17	34.56 (16)
C2—C3—C4—C5	1.8 (3)	C1—S1—C12—C17	150.39 (14)
C9—C3—C4—C5	−177.33 (17)	O2—S1—C12—C13	−152.09 (15)
C2—C3—C4—Br1	−178.05 (12)	C1—S1—C12—C13	−36.26 (17)
C9—C3—C4—Br1	2.9 (2)	C17—C12—C13—C14	−0.5 (3)
C3—C4—C5—C6	−0.3 (3)	S1—C12—C13—C14	−173.54 (14)
Br1—C4—C5—C6	179.50 (13)	C12—C13—C14—C15	0.3 (3)
C3—C4—C5—C10	179.78 (18)	C13—C14—C15—F1	179.50 (17)
Br1—C4—C5—C10	−0.4 (2)	C13—C14—C15—C16	0.0 (3)
C4—C5—C6—C7	−0.9 (3)	F1—C15—C16—C17	−179.61 (17)
C10—C5—C6—C7	179.00 (17)	C14—C15—C16—C17	−0.1 (3)
C5—C6—C7—O1	−179.30 (15)	C15—C16—C17—C12	−0.1 (3)
C5—C6—C7—C2	0.6 (3)	C13—C12—C17—C16	0.4 (3)
C8—O1—C7—C6	179.95 (17)	S1—C12—C17—C16	173.82 (15)

### Hydrogen-bond geometry (Å, °)

**Table:**

*D*—H···*A*	*D*—H	H···*A*	*D*···*A*	*D*—H···*A*
C14—H14···O2^i^	0.95	2.35	3.232 (2)	154

Symmetry codes: (i) *x*−1, *y*, *z*.
